# Mindfulness Training Targets Neurocognitive Mechanisms of Addiction at the Attention-Appraisal-Emotion Interface

**DOI:** 10.3389/fpsyt.2013.00173

**Published:** 2014-01-10

**Authors:** Eric L. Garland, Brett Froeliger, Matthew O. Howard

**Affiliations:** ^1^Supportive Oncology and Survivorship Program, Huntsman Cancer Institute, University of Utah, Salt Lake City, UT, USA; ^2^College of Social Work, University of Utah, Salt Lake City, UT, USA; ^3^Department of Neuroscience, Hollings Cancer Center, Medical University of South Carolina, Charleston, SC, USA; ^4^School of Social Work, University of North Carolina at Chapel Hill, Chapel Hill, NC, USA

**Keywords:** mindfulness, addiction, substance dependence, neurocognitive, stress, reward, automaticity, reappraisal vs. suppression

## Abstract

Prominent neuroscience models suggest that addictive behavior occurs when environmental stressors and drug-relevant cues activate a cycle of cognitive, affective, and psychophysiological mechanisms, including dysregulated interactions between bottom-up and top-down neural processes, that compel the user to seek out and use drugs. Mindfulness-based interventions (MBIs) target pathogenic mechanisms of the risk chain linking stress and addiction. This review describes how MBIs may target neurocognitive mechanisms of addiction at the attention-appraisal-emotion interface. Empirical evidence is presented suggesting that MBIs ameliorate addiction by enhancing cognitive regulation of a number of key processes, including: clarifying cognitive appraisal and modulating negative emotions to reduce perseverative cognition and emotional arousal; enhancing metacognitive awareness to regulate drug-use action schema and decrease addiction attentional bias; promoting extinction learning to uncouple drug-use triggers from conditioned appetitive responses; reducing cue-reactivity and increasing cognitive control over craving; attenuating physiological stress reactivity through parasympathetic activation; and increasing savoring to restore natural reward processing. Treatment and research implications of our neurocognitive framework are presented. We conclude by offering a temporally sequenced description of neurocognitive processes targeted by MBIs through a hypothetical case study. Our neurocognitive framework has implications for the optimization of addiction treatment with MBIs.

## Introduction

Although addiction has been a subject of societal concern for millennia, over the past several decades, discoveries from cognitive and affective neuroscience have deepened our understanding of this age-old, vexing, and pernicious problem. The perspective emerging from several lines of research is one in which addiction is seen as a cycle of compulsive drug-seeking behavior fueled by dysregulated neurocognitive processes (1, 2). Key processes implicated in addiction include motivated attention, automaticity, reward processing, emotion regulation, stress reactivity, and inhibitory control. Studies suggest that these processes arise from individual differences in broadly distributed, functionally- and anatomically-integrated, cortico-limbic-striatal circuits that subserve acquisition, maintenance, and reinstatement of addictive behaviors (3).

Despite these advances in the basic science of addiction, many behavioral interventions for this disorder lag behind the ever-accelerating pace of discovery and have yet to systematically integrate and apply neuroscience findings into treatment development. To the extent that novel psychological therapies target dysregulated neurocognitive processes underlying addictive behavior, they may hold promise as effective treatments for persons with substance use disorders. Mindfulness-based interventions (MBIs) such as Mindfulness-Based Relapse Prevention (4, 5) or Mindfulness-Oriented Recovery Enhancement (6–8) address manifold mechanisms implicated in addiction. An emerging body of controlled trials indicates that MBIs may produce significant therapeutic effects among persons struggling with various forms of addiction, including alcohol (7), drug (4), prescription-opioid (8), and nicotine (9) dependence, among others. Despite early theoretical accounts of the effects of MBIs on information processing in addiction (10), the therapeutic mechanisms of these emerging therapies remain unclear.

This review offers a novel conceptual framework with which to understand how MBIs may ameliorate addiction, with a focus on how such interventions target pathogenic cognitive, affective, and neurobiological mechanisms that contribute to addictive behavior. This framework is first grounded in a brief description of risk chain linking cue-reactivity, implicit cognition, and dysfunctional cognitive control efforts that drives the appetitive motivational states and drug-seeking behaviors characteristic of addiction. Next, we propose a number of hypothetical neurocognitive targets of MBIs, and critique supporting empirical evidence from extant literature on mindfulness and addiction. To promote future research in this area, we recommend behavioral tasks and psychophysiological measures that could be used as mechanistic probes of MBI treatment effects. Finally, we suggest how our neurocognitive framework might lead to optimization of the next generation of MBIs for persons suffering from addictive disorders.

## A Neurocognitive Model of Addiction

### Addictive responses arise from automatic habits and unregulated craving and affect

Research with animals and humans demonstrates that chronic administration of psychoactive drugs results in adaptations in multiple neurotransmitter systems in the brain, consequentially altering functional neural circuitry that governs a broad array of interactive processes (e.g., affect and reward, habit learning and memory, and cognitive control over prepotent environmental stimuli). Though the neurobiological bases of the effects of chronic drug abuse on brain-behavior relations remain to be fully elucidated, current research suggests that the dopamine system plays a critical role in the progression from drug initiation to chronic and habitual self-administration. Psychoactive drug-use induces dopaminergic activity in the ventral striatum and ventral tegmental area (11), resulting in a robust form of positive reinforcement. However, following chronic use, drug administration effects on dopamine neurotransmission are attenuated in the ventral striatal-reward pathway and potentiated in the dorsal striatal-habit learning pathway (12).

Repeated use of psychoactive substances is believed to impart motivational significance to cues associated with drug-use episodes through sensitization of mesocorticolimbic brain regions (13). As such, drug-related cues come to evoke powerful, conditioned motivational responses that may be fully dissociable from the pleasure and reward elicited by drug use (14, 15).

This conditioned response to drug-related cues (i.e., cue-reactivity), manifests as a constellation of somatic sensations coupled with a broad array of physiological reactions including autonomic, corticolimbic, corticostriatal, and neuroendocrine responses (16–19). The habitual behavioral response to drug-related cues is thought to be coordinated by drug-use action schemas, i.e., memory systems that drive drug seeking and drug use through automatized sequences of stimulus-bound, context-dependent behavior (20, 21). Importantly, cue-reactivity confers compulsivity to drug-seeking behaviors, motivating the addict to consume drugs even after long periods of abstinence and despite countervailing motivations to remain abstinent, particularly in contexts that elicit unregulated stress and negative affect (1). Indeed, stress biases behavior toward habitual responding (22).

Because obtaining and consuming psychoactive substances are motivationally salient goals in addiction, drug-use action schemas stored in memory guide implicit cognitive processing of stimuli associated with previous drug-use episodes. This implicit cognitive process is manifested as a preferential focus of attention toward drug-related cues, known as addiction attentional bias (23). When attention is focused on drug-related cues, motivation for drug-use increases, which then amplifies the salience of the cues (24). Thus, addiction attentional bias and craving are mutually excitatory processes (25) that can compel drug-use even in the absence of the volition or intent to use drugs. As such, an addict may find him or herself consuming drugs without awareness of the intention to use, in much the same way other complex goal-oriented, thought-action repertoires can be engaged habitually without conscious volition by conditioned contextual cues (26).

### Dysregulation of context-dependent prefrontally mediated control in addiction

As the addictive habit becomes more entrenched, individuals struggling with addiction experience a loss of cognitive and behavioral control. Cognitive control deficits include those affecting attentional and inhibitory control (27) and deficits in cognitive regulation of stress and affect (1). Functional neuroanatomical correlates of the effects of substance abuse include alterations in cingulate and prefrontal cortices (PFCs), two brain structures crucial for error monitoring (28) and inhibitory control, respectively (29). For example, compared to healthy individuals, persons with substance use disorders exhibit hypoactivation in frontal cognitive control circuitry during inhibition of cognitive interference and conflict resolution (30, 31), as well as during processing of salient emotional information (31–35). Acute withdrawal further dysregulates prefrontal brain function across an array of cognitive tasks (36–41). Thus, as cognitive control over behavior becomes impaired due to neurocognitive changes that occur with the development of addiction, the addict progressively loses control over the addictive habit.

As a result of these cognitive control deficits, actively addicted persons may experience an intense, overwhelming compulsion and motivation to seek and use drugs that is difficult to regulate. Moreover, persons in recovery from addiction often experience the impulse to use substances as intrusive and incongruent with their desire to remain abstinent (42). Yet, due to deficient or atrophied proactive cognitive control, such individuals may attempt to reactively inhibit this upwelling of unbidden motivational drive by employing “willpower” to suppress the urge to engage in the addictive behavior. Thus the pendulum of prefrontal regulation swings from a context of under-control to one of over-control. Through such over-control, suppression may promote relapse insofar as this cognitive strategy inadvertently results in a “rebound effect,” i.e., an increased rate of the thoughts and emotions it is directed against (43, 44). When attention is deployed in search of undesirable mental content to be suppressed (e.g., a drug craving, negative affect), the ensuing positive feedback loop leads to hyperaccessibility of unwanted cognitions (45), amplifying their frequency and intensity under conditions of stress (46). Consistent with these deleterious effects of suppression on resolving emotional conflict, neuroimaging research demonstrates that unlike reappraisal of negative emotion which involves potentiated prefrontal response coupled with attenuated amygdala response (47, 48), suppression shifts the time-course of prefrontal response (i.e., delays) while potentiating amygdala response to negative emotional information (48). Furthermore, individuals who rely on suppression as a regulatory strategy exhibit greater amygdala activation in response to negative emotional information than individuals who tend to use reappraisal (49). Thus, while hypoactivation in cognitive control circuits may result in an inability to effectively inhibit automatized addictive responses, excessive and ill-timed hyper-activation of frontal-executive resources during suppression of negative emotions and urges may also fail to resolve emotional conflict. In contrast to context-dependent forms of prefrontal regulation that sensitively accommodate extant cognitive contexts to challenging mental contents (i.e., reappraisal), the use of suppression to rid oneself of craving or affective states once they have already arisen is costly in terms of neurocognitive resources and likely to fail.

This failure to resolve conflict between emotional drives (e.g., craving) and higher-order goals (e.g., the goal of abstinence) during attempted suppression may further bias cognitive processing toward drug-related cues and mental contents, inadvertently increasing attention to substance-related thoughts and urges (50, 51). Consequently, suppression of thoughts of substance use leads to greater enactment of consummatory behaviors (52, 53). When addictive urges are chronically suppressed over time, the neurocognitive resources for self-regulation are depleted, resulting in an inability to inhibit substance-related cognitions and an attentional bias toward drug-related cues (54). Ultimately, regulatory resource depletion that occurs during sustained suppression of urges may contribute to relapse.

### Summary of the neurocognitive model of addiction

**Figure 1:**
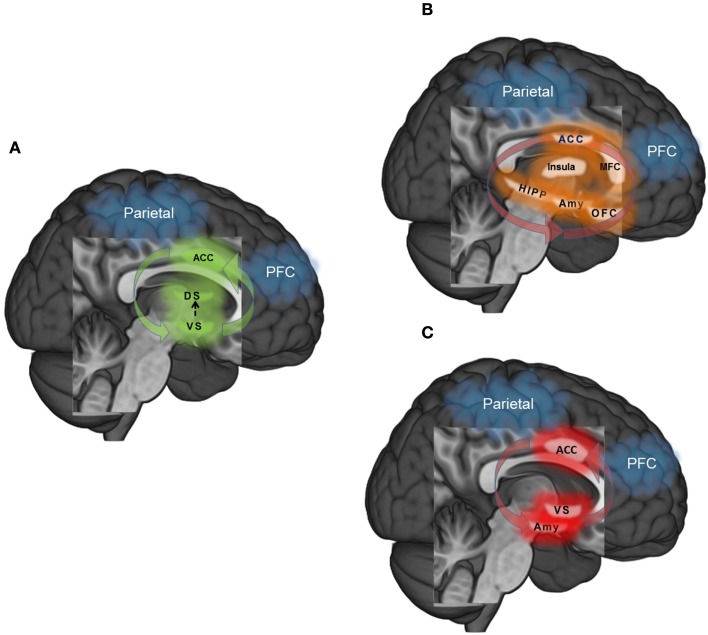
**(A)** Habit – automaticity: learning to approach rewarding stimuli (i.e., psychoactive drugs) is reinforced via dopaminergic innervation in the ventral striatum (VS). However, as the association between stimulus and reward becomes learned, memory and motor-related commands transition to dorsal striatum (DS). In the context of overlearned and automatized behavior such as drug addiction, fronto-parietal attentional networks that exert top-down modulatory control over behavior may become functionally disconnected with behavior. **(B)** Unregulated craving: drug craving is a multi-dimensional process that is elicited by internal and external triggers. Cues that trigger craving may elicit greater response in ACC during attentional monitoring, medial frontal cortex (MFC) during appraisal of the salience of the cue, amygdala (Amy) during emotional arousal, hippocampus (HIPP) during coding of context, insula during interoception and orbitofrontal cortex (OFC) in weighing the cost – benefit ratio of relieving craving through drug use or remaining abstinent. Functional disconnectivity of the fronto-parietal network with these regions may strengthen craving by dysregulating responses to craving triggers. **(C)** Unregulated affect: Dysregulated affect may ensue when there is inefficient or attenuated top-down control from fronto-parietal circuitry into the amygdala (Amy) in the context of negative emotions, and ventral striatum (VS) in the context of reward. Disconnectivity between the anterior cingulate cortex (ACC) and prefrontal cortex along with hyperconnectivity of the ACC and limbic-striatal regions may potentiate this dysfunction.

**Figure 2:**
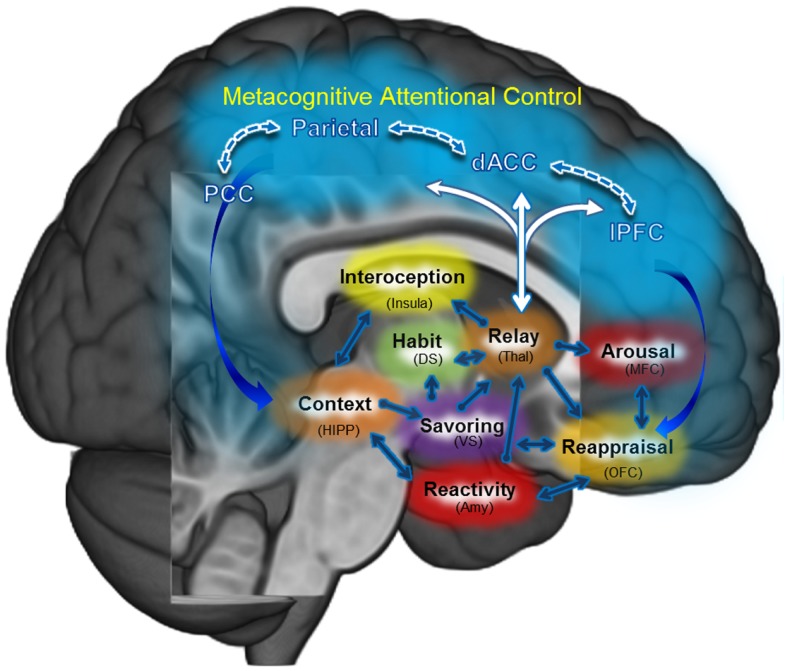
**Mindfulness-centered regulation: the central tenet of this model posits that mindfulness-based interventions (MBI’s) may remediate dysregulated habit behaviors, craving, and affect primarily by way of strengthening functional connectivity: (1) within a metacognitive attentional control network (PFC, ACC, Parietal); and (2) between that metacognitive attentional control network and the (a) habit circuit, (b) craving circuit, and (c) affect circuit**.

In sum, the maintenance of drug addiction is a manifold process, putatively subserved via dysregulation within and between multiple, dynamic neurocognitive processes. Though not an exhaustive model of the time-course of addiction, we focus here on three primary systems where neuroscience models of addiction from the extant literature converge to shed light on dysregulated behavioral control: (1) habit responding-automaticity (Figure 1A), (2) unregulated craving (Figure 1B), and (3) unregulated affect (Figure 1C). First, habitual or automatized drug-taking behavior is related to transfer from ventral to dorsal striatal mediation of motor commands, hyperactivity in ACC in response to drug-related cues, and weakened functional connectivity between a PFC-parietal attention network and striatal circuitry. Secondly, unregulated craving may result from disrupted feedback from the fronto-parietal attention network to broadly distributed yet highly interconnected circuits involved in contextual learning (hippocampus), interoception (insula), emotional reactivity and conditioning (amygdala), appraisal of emotionally salient stimuli (medial frontal cortex: MFC), emotion regulation and decision making (orbital-frontal cortex: OFC), and attention and conflict resolution (dorsal anterior cingulate cortex; dACC). Thirdly, unregulated affect may similarly result from dysfunctional fronto-parietal network feedback response, but in this context, ineffectively modulating amygdala reactivity to negative emotional information and ventral striatum (nucleus accumbens) during reward processing, coupled with aberrant ACC response. Common to all three dysregulated neural circuits are: (1) hyper-involvement of the dACC in the dysfunctional process, and (2) hypo-involvement of a fronto-parietal attention network necessary for exerting top-down regulation.

Though dysregulation in each circuit likely represents both a vulnerability for transitioning from casual drug use to addiction and a consequence of long-term use, investigating the effects of each form of neural dysfunction, and interactions between them, will shed new light on the multiple pathways by which addiction reinforces maladaptive (drug-taking) behavior. From this neurocognitive perspective, addiction occurs through basic human learning processes gone awry; neural resources devoted to learning become hijacked due to the neuropharmacologically rewarding properties of the substance (55). Once an intentional decision, over time the act of seeking and consuming drugs is established as an automatic, compulsive habit, one that becomes increasingly difficult to inhibit as brain structures involved in self-regulation become dysregulated by the combined action of stress and the pharmacologic agent itself. Unwittingly, the struggle to reassert control over the addictive behavior through misguided attempts at urge suppression results in hypervigilance for salient cues such as the sight of a bar, an old “hang-out” spot, or a familiar “drinking buddy,” which trigger uncomfortable physical sensations and a strong desire to consume substances, even after extended periods of abstinence. Eventually, the addict succumbs and relapses, which strengthens the addictive habit through the processes of conditioning and negative reinforcement (56). Hence, behavioral interventions that aim to interrupt automatized drug-use action schemas and restore more normalized reward learning processes may prove to be beneficial in helping drug abusers maintain abstinence. Similarly, treatment approaches that train context-dependent prefrontally mediated cognitive control as an alternative to the maladaptive strategy of suppressing addictive urges may free neurocognitive resources for the effective regulation of emotional distress and substance craving. It is in these regards that MBIs may be especially efficacious for the treatment of addiction.

## Mindfulness Training Ameliorates Addiction by Targeting Neurocognitive Mechanisms

Although mindfulness is an English term linked with a set of contemplative practices and principles originating in Asia over 2500 years ago, in its modern usage, mindfulness refers to a psychological phenomenon currently being studied for its relevance to mental and physical health in fields such as medicine, psychology, and neuroscience. Across these fields, a body of literature has accrued supporting the efficacy of MBIs for a range of biobehavioral disorders, including but not limited to addiction. Indeed, there is support for the effectiveness of MBIs in reducing stress and improving clinical outcomes across disorders as diverse as depression (57), irritable bowel syndrome (58), and chronic pain (59). Consequently, MBIs are increasingly well-regarded for their therapeutic promise.

MBIs are centered on practices designed to evoke the *state* of mindfulness, a mindset characterized by an attentive and non-judgmental metacognitive monitoring of moment-by-moment cognition, emotion, perception, and sensation without fixation on thoughts of past and future (60, 61). The *practice* of mindfulness involves two primary components: focused attention and open monitoring (61, 62). During focused attention, attention is sustained on an object while the practitioner alternately acknowledges and lets go of distracting thoughts and emotions. Objects of focused attention practice can include the sensation of breathing; the sensation of walking; interoceptive and proprioceptive feedback about the body’s internal state, movement, and position; and visual stimuli (e.g., a candle flame) (63).

Focused attention practices are often the precursor to open monitoring forms of mindfulness meditation. During open monitoring, a state of metacognitive awareness is cultivated wherein mental contents are allowed to arise unperturbed without suppression or distraction while the quality of awareness itself remains the primary focus of attention (61). This state of awareness is metacognitive in the sense that it involves monitoring the content of consciousness while reflecting back upon the process or quality of consciousness itself. In other words, the practitioner maintains awareness of the locus of attention (without trying to retain focus on a particular object) and his or her level of cognitive arousal without reacting to or elaborating on any particular content of consciousness, which, from this mental stance, are viewed as insubstantial and ephemeral. Putatively, focused attention and open monitoring emphasize or differentially activate different cognitive capacities during the mindful state, including attentional vigilance, attentional re-orienting, executive monitoring of working memory, response inhibition, and emotion regulation (62). As such, they are often combined during a single practice session, which typically commences with focused attention and then evolves toward a more open monitoring approach.

Engaging in these practices repeatedly over time may induce neural and cognitive plasticity (7); recurrent activation of the mindful state during meditation may leave lasting neurobiological traces that accrue into durable changes in the dispositional propensity to be mindful in everyday life even while not meditating (64). In that regard, MBIs can produce significant increases in dispositional mindfulness that mediate the effects of mindfulness training on clinical outcomes (65). Germane to the current discussion of neurocognition in addiction, dispositional mindfulness is significantly inversely associated with addiction attentional bias (1) and craving (66), positively associated with autonomic recovery from stress and substance cue-exposure (67), and correlated with various indices of cognitive control (68–70). MBI-related increases in dispositional mindfulness might be mediated through neuroplasticity stimulated by experience-dependent alterations in gene expression (71, 72). Indeed, cross-sectional studies have demonstrated significant differences in gray matter volume between meditation practitioners and meditation-naïve controls, particularly in regions of PFC that instantiate cognitive control (e.g., inferior frontal gyri) and higher-order associative processing (e.g., hippocampus) (73–77). Moreover, longitudinal research has shown that participants in an 8-week MBI evidenced increased gray matter density in posterior cingulate cortex, temporo-parietal junction, and cerebellum, compared to controls (78), and reduced amygdala volume that correlated with the degree of stress-reduction achieved from mindfulness training (79).

Through focused attention and open monitoring forms of meditation, MBIs exercise a number of neurocognitive processes believed to go awry in addiction. Indeed, MBIs may be fruitfully conceptualized as means of training or exercising prefrontally mediated cognitive control networks which have become atrophied or usurped in the service of drug seeking and use. By strengthening PFC functions and the ability of the PFC to modulate other brain networks in a context-dependent manner, MBIs may provide the global benefit of enhancing neurocognitive flexibility – augmenting a “domain-general” resource that may then be applied across the manifold subcomponent processes implicated in psychological health (e.g., cognitive regulation of automaticity, attention, appraisal, emotion, urges, stress reactivity, reward processing, and extinction learning). These processes do not operate in isolation; they are linked in mutually interdependent, interpenetrating, recursive networks [for reviews, see Ref. (2, 3)]. MBIs may restructure dysregulated processes by strengthening functional connectivity and efficiency of prefrontally mediated self-regulatory circuits (see Figure 2). Below, we propose a number of hypothetical neurocognitive targets that could mediate the therapeutic effect of MBIs on addictive behavior.

### Habit responses

Substance dependent individuals typically experience euphoria during initial stages of drug-use. Yet, as experience with the drug increases, the reward associated with drug-taking becomes dramatically attenuated. Despite diminishing returns in positive emotional experiences resulting from substance use, dependent users continue to use their drug of addiction. Undergirded by neuroplastic changes in striatal circuitry, habitual drug-use becomes an overlearned process that can become automatized (12, 80).

Relatedly, human positron emission tomography (PET) research has found that meditation practice increases dopamine release in the ventral striatum (81). This pioneering study by Kjaer et al. (81) suggests that MBIs may target striatal-dopamine transmission – a neural function believed to mediate automaticity that becomes dysregulated following chronic drug-use. Though more investigation is needed to elucidate effects of mindfulness on brain-behavior relations subserving drug-use action schemas, early research on the effects of mindfulness on behavioral measures of automaticity has emerged [e.g., Ref. (82)]. Such research provides a theoretical foundation for the potential efficacy of MBIs for interrupting drug-use action schemas. Hypothetically, mindfulness training may increase awareness of the activation of drug-use action schemas when triggered by substance-related cues or negative emotion, thereby allowing for the disruption of automatized appetitive processes with a controlled coping response. As posited in our model of mindfulness-centered regulation (Figure 2), mindfulness training may enhance functional connectivity in a cortico-thalamic loop including prefrontal, cingulate, parietal, and dorsal thalamus nodes, strengthening an executive regulatory circuit providing feedback to the striatum and medial temporal lobe. This feedback process is theorized to allow for greater consciousness of thoughts and behaviors that were previously enacted with little conscious awareness.

The practice of mindfulness in daily life is focused on developing awareness of automatic behavior. Indeed, many MBIs prescribe informal mindfulness practices where individuals are instructed to engage in everyday, repetitive tasks (e.g., washing the dishes) with full consciousness of the sensorimotor aspects of the activity. Such informal mindfulness practices are designed to reduce mind-wandering and strengthen conscious control over automaticity. Potentially as a result of such practices, mindfulness training has been shown to decrease habit behavior (83) and reduce rigid adherence to scripted cognitive responses (82). These findings accord with early theoretical accounts which conceptualized mindfulness meditation as a form of “deautomatization,” whereby patterns of motor and perceptual responses which had been rendered automatic and unconscious through repetition are reinvested with conscious attention (84). Thus, is plausible that mindfulness training may deautomatize habitual addictive responses through both formal meditations focused on regulating automatic appetitive impulses as well as informal mindfulness practices designed to increase generalized awareness of automaticity. In light of findings suggesting that conscious cognitive control disrupts automatic processing (20, 85–87), mindfulness training may interrupt drug-use action schemas by augmenting top-down control via a frontoparietal metacognitive attention network, facilitating the strategic deployment of self-regulatory processes to reduce or prevent substance use. The effects of mindfulness training on inhibition of habit responses might be indexed with performance on an Emotional GoNoGo task (88), where subjects would be asked to withhold automatized “go” responses in the context of emotional interference from a drug-related (i.e., a drug-related background image) or negative affective stimulus (i.e., an aversive background image).

### Attentional bias

Given that drug-use action schemas may be evoked by cues associated with past substance use episodes, activation of addictive habits may be interrupted by re-orienting attention from substance-related stimuli to neutral or salutary objects and events. MBIs may be especially efficacious in that regard. Focused attention and open monitoring mindfulness practices capitalize on attentional orienting, alerting, and conflict monitoring – the fundamental components of attentional control (89, 90). Consequently, studies indicate that mindfulness is linked with enhanced attention regulation (61, 91). For instance, mindfulness training is associated with strengthening of functional connectivity within a dorsal attentional network (92) and MBIs can increase attentional re-orienting capacity, i.e., the ability to engage, disengage, and shift attention efficiently from one object to another subserved by dorsal attentional systems (93, 94). Other studies demonstrate that long-term mindfulness training strengthens alerting (93, 95), i.e., a vigilant preparedness to detect and attend to incoming stimuli, subserved by the ventral attentional stream. In addition, dispositional mindfulness is positively associated with self-reported attentional control (68) and behavioral indices of sustained attention capacity (70). Recently, data from a randomized controlled trial indicated that 8 weeks of Mindfulness-Oriented Recovery Enhancement led to significant reductions in attentional bias to pain-related cues in a sample of opioid-misusing chronic pain patients (96).

MBIs may target addiction attentional bias by facilitating attentional disengagement from substance-related stimuli. In support of this hypothesis, a study of alcohol dependent adults in residential treatment identified a significant negative correlation between dispositional mindfulness and alcohol attentional bias for stimuli presented for 2000 ms that remained robust even after controlling for alcohol dependence severity, craving, and perceived stress (1). Hypothetically, alcohol dependent persons higher in dispositional mindfulness might exhibit increased capacity for attentional disengagement from alcohol cues by virtue of enhanced PFC and anterior cingulate cortex functionality, as these brain structures have been implicated in addiction attentional bias (97–99). Concomitantly, the degree to which alcohol dependent individuals higher in dispositional mindfulness were better able to disengage their attention from alcohol cues than their less mindful counterparts predicted the extent of heart-rate variability (HRV) recovery (an index of prefrontal-autonomic regulation) from stress-primed alcohol cue-exposure (67). Mindfulness training may also affect attentional orienting to substance-related cues. Among a sample of alcohol dependent adults in inpatient treatment, Mindfulness-Oriented Recovery Enhancement was found to result in significant effects on alcohol attentional bias for cues presented for 200 ms (7), indicating modulation of automatic initial orienting to alcohol cues [c.f. (23)]. In individual difference analyses, reductions in attentional bias following Mindfulness-Oriented Recovery Enhancement were significantly associated with decreases in thought suppression, which were, in turn, correlated with increases in HRV recovery from alcohol cue-exposure and improvements in self-reported ability to regulate alcohol urges.

Hence, mindfulness training may strengthen the capacity to regulate attention in the face of conditioned stimuli associated with past substance use, countering attentional biases by refocusing attention on neutral or health-promoting stimuli (e.g., the sensation of one’s own breath or a beautiful sunset). Repeatedly redirecting attention from substance-related cues toward innocuous stimuli may foster extinction of associations between substance-related cues and drug-use action schema. This potential mechanism may explain how attentional bias modification among addicts leads to decreased substance use and improved treatment outcomes (100, 101). Future research could evaluate the effects of mindfulness training and MBIs on addiction attentional bias with the use of a dot probe task alone or coupled with eye tracking and analysis of event-related potentials (ERPs) to determine at what stage of attentional selection (initial orienting vs. later attentional disengagement) training has significant effects.

### Cue-elicited craving

The urge to seek intoxication from addictive substances is driven, in part, by reactivity to substance-related stimuli which have been conferred incentive salience, and is magnified by negative affective states. Several studies demonstrate that MBIs can produce significant reductions in craving (4, 8, 102–105). However, other studies have failed to identify significant reductions in craving among participants of MBIs (7, 106–108).

Mindfulness-based interventions may positively influence craving-related processes in several ways. First, mindfulness training may decrease bottom-up reactivity to drug-related stimuli, as mediated by reduced activation in the subgenual anterior cingulate cortex and striatum during exposure to substance cues (105). Second, mindfulness training may decouple negative emotion from craving. Although negative emotion is a common precipitant of craving and subsequent relapse (109), mindfulness training may extinguish this association, such that an addict experiencing sadness, fear, or anger could allow these emotions to arise and pass without triggering an appetitive reaction. Indeed, substance dependent individuals participating in Mindfulness-Based Relapse Prevention were less likely to experience craving in response to depressed mood, and this reduced craving and reactivity to negative emotion predicted fewer days of substance use (110).

MBIs may also produce therapeutic effects by increasing awareness of implicit craving responses. Tiffany (20) proposed that conscious craving occurs when an activated drug-use action schema is blocked from obtaining the goal of drug consumption. As such, persons in acute withdrawal, persons unable to obtain drugs (e.g., due to lack of funds or availability), or persons attempting to maintain abstinence in the face of triggers may experience an upwelling of craving for substances. In contrast, according to this theory, addicts who are able to obtain and use drugs in an unimpeded fashion would not experience craving. Similarly, persons in long-term residential treatment who are isolated from drug-related cues are unlikely to be conscious of craving. Without awareness of craving, the addict may unwittingly remain in high-risk situations and thus be especially subject to relapse. Indeed, lack of awareness of substance craving has been shown to be predictive of future relapse (111). MBIs may increase conscious access to the appetitive drive to use substances by virtue of their effects on increasing interoceptive awareness (78, 112). In that regard, mindfulness training has been shown to increase activity in the anterior insula during provocations by emotionally salient stimuli (113, 114). The anterior insula subserves interoception and awareness of the physical condition of the body, among other related processes (115). Increased neural activity in the insula during mindfulness meditation may index heightened access to interoceptive information.

In synthesizing the findings regarding attentional bias and cue-induced craving, we suggest that MBIs may restructure attentional bias away from drug-related reinforcing stimuli (e.g., drug-cues, negative affective stimuli) and facilitate the addict’s attempts to deal with associated cravings. We posit that mindfulness-centered regulation of cue-elicited appetitive responses occurs as a result of strengthening frontal-executive circuit-function and enhancing neural communication to the hippocampus and thalamus through formal and informal mindfulness meditation practices. The hippocampus is critical for context-dependent learning and memory – with reciprocal connectivity to brain regions that code for reward (ventral striatum), interoception (insula), affect (amygdala), and thalamus. In turn, the thalamus, a complex structure that is generally considered to serve as a relay station between limbic, striatal, and cortical circuits, contains efferent and afferent projections with striatal, limbic, somatosensory, ACC, lateral and medial PFC, and OFC. Thus, the recovering addict may utilize mindfulness training to become aware of *which* cues are under the spotlight of attention, and become more sensitive to *how* those cues may trigger changes in body state and motivation drive.

Hence, mindfulness may increase awareness of craving and thereby facilitate cognitive control of otherwise automatic appetitive impulses. In that regard, a recent study found that participation in Mindfulness-Oriented Recovery Enhancement was associated with decreased correlation strength between opioid craving and opioid misuse, suggesting that mindfulness training may have decoupled appetitive responses from addictive behaviors (8). This mechanism may explain the disparate findings vis-a-vis the effects of mindfulness on craving: because of potential underreporting of baseline levels of craving among individuals with impaired insight into their addiction (34), this increased awareness may confound researchers’ attempts to measure the impact of mindfulness training on craving, resulting in an apparent lack of change in craving over time.

The effects of mindfulness on cognitive regulation of craving might be measured by utilizing neuroimaging methodology (e.g., fMRI) to investigate neural circuitry function while participants attempt to regulate their craving response to salient drug-cues. For example, cognitive regulation appears to decrease cigarette craving concomitant with increased activity in dACC (116) and prefrontal regions coupled with attenuated activity in striatal regions (117). A complementary approach to probing the effects of mindfulness on regulating craving may be to utilize real-time fMRI (rt-fMRI). rt-FMRI involves providing subjects with real-time feedback of the BOLD signal within a brain region of interest (ROI) while they attempt to regulate the response within that ROI. This approach has been used to manage pain (118) and reduce cigarette cue craving in nicotine dependent smokers during smoking cessation (119). Evaluating the effects of mindfulness-centered regulation of craving-related neural circuitry in real-time may include a number of benefits including: (a) directly measuring which circuits are being effectively modulated and which are not; (b) feedback to the subject that will help guide mindfulness efforts; and (c) identifying individual differences associated with differential effects of MBIs on specific neural mechanisms.

### Cognitive appraisal

Insofar as stress evokes automatic responses and impairs prefrontally mediated cognitive control functions (120), exposure to socioenvironmental stressors may render addicts in recovery vulnerable to relapse (1, 22, 121). Mindfulness training may allay stress-induced relapse by virtue of its stress-reductive effects (122). Although early theorists believed that mindfulness meditation reduced stress primarily by evoking a generalized relaxation response (123), modern research indicates that mindfulness practice may also attenuate stress by targeting cognitive mechanisms (1, 124). One potential target of mindfulness is cognitive appraisal, the process whereby stimuli and their environmental context are evaluated for their significance to the self (125). Appraisals of threat or harm elicit negative emotional reactions coupled with activation of stress physiology. When recurrent, such emotional reactivity biases perception, leading to exaggerated, overestimated appraisals of threat and underestimations of self-efficacy (126), and ultimately, sensitization to future stressors (127).

In contrast, mindfulness, which has been conceptualized as a non-reactive form of awareness (128) may enable the individual to cognitively appraise his or her present circumstances with less emotional bias, and to more accurately assess his or her ability to cope with present challenges (60). Thus, MBIs may impact both primary (rapid and implicit) and secondary (slow and explicit) appraisal processes (125). In partial support of this hypothesis, a recent neuroimaging study revealed that, in contrast to a meditation-naive control group, mindfulness meditation practitioners exhibited decreased reactivity to briefly presented negative emotional cues in frontal-executive brain regions (i.e., dorsolateral PFC) and less deterioration of positive affect in response to cue-elicited amygdala activation (31). These data suggest that mindfulness training may alter the allocation of cognitive resources during appraisal of negative emotional stimuli and attenuate the influence of limbic reactivity on mood state. Other research demonstrates that mindfulness training minimizes emotional interference from unpleasant stimuli [e.g., Ref. (129)]. In so doing, mindfulness training may reduce biases toward negative emotional information processing. Among persons with a history of depression, Mindfulness-Based Cognitive Therapy reduces overgeneral memories (130) and cognitive bias toward negative information (131). Among individuals suffering from chronic pain, Mindfulness-Oriented Recovery Enhancement decreases cognitive bias toward pain-related cues (96). Together, these findings suggest that MBIs may decrease negative emotional bias in initial cognitive appraisal processes, thereby reducing the downstream effects of stress on addictive behavior. As mindfulness-centered regulation enhances cortico-thalamic-limbic functional connectivity, the recovering addict becomes more aware of relations between attention, emotional state, and motivation. This awareness provides an opportunity to deploy cognitive strategies to respond to the environment in a more adaptable context-dependent manner, rather than responding from a pattern of overlearned reactive behaviors.

One approach to evaluating the effects of mindfulness-centered regulation of stress appraisal may be to utilize fMRI paradigms like the affective Stroop task to probe cognition-emotion interactions (31). If mindfulness reduces stress appraisals, aversive stimuli may produce less emotional interference during cognitive task performance, resulting in reduced reaction time decrements and decreased activity in prefrontal-limbic circuitry on trials following aversive cues. Alternatively, phasic cortisol output and sympathetic nervous system reactivity could be measured during laboratory stress induction techniques such as the Trier Social Stress Test (132) and correlated with self-reported appraisals of stress versus challenge during the task both pre- and post-mindfulness training.

### Emotion regulation

When individuals are unable to marshal effective problem-solving to resolve a stressor, lack of a favorable resolution may lead to deployment of emotion regulation efforts to manage the emotional distress elicited by the stressful circumstance. Neuroimaging research has provided evidence for a reciprocal, dual-system neural network model of emotion regulation comprised of a dorsal brain system (e.g., dlPFC, dACC, parietal cortex) subserving top-down cognitive control, and a ventral brain system (e.g., amygdala, striatum) subserving bottom-up emotional impulses (133–135). Top-down engagement of proactive cognitive control mechanisms regulates negative affect and attenuates the effects of emotional interference on cognition (135–138), and is associated with increased activation of PFC which in turn attenuates amygdala activation (139, 140). Research suggests that dysregulated emotional reactions occur when the reciprocal balance between the relative activation of bottom-up and top-down neural circuits becomes tipped in favor of bottom-up processes (141). A number of studies suggest that mindfulness training may counter this imbalance and augment emotion regulation [for reviews, see Ref. (78, 142)] by restructuring neural function in favor of context-dependent top-down control processes. For example, Goldin and Gross (143) demonstrated that individuals with elevated negative affect at baseline who later received mindfulness training exhibited increased emotion regulatory capacity coupled with greater recruitment of attentional control resources and reduced amygdala activation during exposure to negative, self-relevant stimuli. Thus, by enhancing top-down cognitive control over emotional responses in a context-dependent fashion, MBIs may reduce drug use precipitated by negative affective states.

Importantly, MBIs provide training in cultivating a state of mindful awareness and acceptance of the extant emotional response as a precondition for emotion regulation. While acceptance of aversive mental experience may itself result in reduced negative affect (144), mindfulness training may also exert downstream facilitative effects on cognitive regulation of emotion following the acute state of mindfulness. For instance, mindfulness training may promote cognitive reappraisal, the process by which the meaning of a stressful or adverse event is re-construed so as to reduce its negative emotional impact (125). One theoretical model posits a multi-stage process of mindful emotion regulation (1, 145). According to this model, during an adverse experience mindfulness practitioners first disengage from initial negative appraisals into the metacognitive state of mindfulness in which cognitions and emotions are viewed and accepted as transitory mental events without inherent veridicality. Subsequently, the scope of attention broadens to encompass a larger set of previously unattended information from which new situational appraisals may be generated. By accessing this enlarged set of contextual data, present circumstances may be reappraised in an adaptive fashion that promotes positive affect and behavioral activation. For instance, a newly abstinent alcohol dependent individual might reappraise an affront by a former “drinking buddy” as evidence of their need to build new, sober relationships. In support of this model, recent studies indicate that mindfulness during meditation predicts enhanced cognitive reappraisal (146), which in turn mediates the association of mindfulness and reduced substance craving (147). This context-dependent use of prefrontal regulatory strategy represents a “middle way” between hypo- and hyper-activation of cognitive control resources, thereby preventing resource depletion and untoward rebound effects.

Speculatively, this “mindful reappraisal” process may involve spreading activation in a number of brain networks. Generating the state of mindfulness in the midst of a negative affective state may activate the ACC and dlPFC (148, 149), which could facilitate metacognitive monitoring of emotional reactivity, foster attentional disengagement from negative appraisals, and regulate limbic activation. In so doing, the acute state of mindfulness may attenuate activation in brain areas that subserve self-referential, linguistic processing during emotional experience (e.g., mPFC) while promoting interoceptive recovery from negative appraisals by increasing activation in the insula (113). Metacognitive disengagement from the initial negative appraisal may result in non-elaborative attention to somatosensory information, thereby facilitating the set shifting process of cognitive reappraisal, as brain activations shift from posterior to anterior regions of cortex centered on the node of the OFC. During this process emotional interference is attenuated while alternate appraisals are retrieved from memory and evaluated for goodness-of-fit to situational parameters and demands (150).

The effects of mindfulness-centered regulation of negative emotion might be measured with a standard emotion regulation paradigm [c.f. (137)], in which participants are instructed to use reappraisal to reduce negative affect in response to exposure to aversive visual stimuli [e.g., images from the International Affective Picture System; (151)]. In this task paradigm, mindfulness practitioners may exhibit enhanced reappraisal efficacy, as evidenced by reduced self-reported and psychophysiological responses to aversive stimuli on reappraise relative to attend trials. In that regard, a study employing ERP analysis found that when compared to controls, meditators exhibited significantly greater reappraisal efficacy as evidenced by significantly larger attenuation of brain activity during reappraisal of stressful stimuli in centro-parietal regions subserving attentional and emotional processing (152).

### Stress reactivity

In addition to pro-regulatory effects on emotion, mindfulness training may facilitate neurocognitive regulation of the effects of stress on the autonomic nervous system. As addicts in treatment develop dispositional mindfulness through mindfulness training, they may be more able to engage prefrontal cortical modulation of the sympathetic “fight-or-flight” response via parasympathetic nervous system activation of the “vagal brake,” resulting in increased HRV and heart-rate deceleration in the face of stress or addictive cues (153, 154). Thus, increasing dispositional mindfulness may be reflective of greater neurovisceral integration and flexibility in the central autonomic network (67). This network is comprised of neuroanatomic and functional linkages between central (e.g., PFC and ACC) and autonomic (e.g., vagus nerve) nervous system structures which coordinate the self-regulation of attention, cognition, and emotion while exerting regulatory influences over perturbations to visceral homeostasis (155), such as those that might be evoked in abstinent substance dependent individuals exposed to stressful and/or substance-related stimuli. Mindful individuals may have greater capacity for contextually appropriate engagement and subsequent disengagement of neurocognitive resources in response to the presence and absence of stress and drug-cues. Such autonomic flexibility (156) engendered through mindfulness training may help persons in recovery from addiction adapt to situational demands without succumbing to a stress-precipitated relapse.

This hypothesis is consistent with evidence of the effects of mindfulness on neural function in dlPFC and ACC (149, 157), key structures involved in central autonomic regulation of HRV via downstream influences on the amygdala and hypothalamus (158, 159). Congruent with such findings, MBIs increase parasympathetically mediated HRV to an even greater extent than relaxation therapy (160, 161), and decreases sympathetically mediated indices of stress (8), including blood pressure (162), heart rate (163), skin conductance responses (161), and muscle tension (164). These effects of mindfulness-centered regulation on autonomic function may result in improved ability to manage substance cue-reactivity. In support of this hypothesis, a Mindfulness-Oriented Recovery Enhancement intervention for alcohol dependence increased HRV recovery from stress and alcohol cue-reactivity (7). Congruent with this finding, relative to their less mindful counterparts, alcohol dependent individuals with higher levels of dispositional mindfulness exhibited greater attentional disengagement from alcohol cues which predicted the extent to which their HRV recovered from alcohol cue-exposure levels (67). Lastly, persons participating a mindfulness-based smoking cessation intervention who exhibited increased HRV during mindfulness meditation smoked fewer cigarettes following treatment than those who exhibited decreased HRV (165). Thus, addicts who develop dispositional mindfulness through participation in MBIs may become better able to regulate appetitive responses by virtue of enhanced neurocognitive control over autonomic reactivity to stress and substance cues.

The effects of MBIs on cognitive regulation of autonomic cue-reactivity might be measured with a stress-primed cue-reactivity paradigm, in which participants are first exposed to a laboratory stress induction [e.g., aversive IAPS images, c.f. (7); or the TSST, c.f. (132)], then exposed to substance-related cues (either *in vivo*, imaginally, or images of alcohol or drugs), and finally asked to use mindfulness skills to downregulate the resultant state of autonomic arousal.

### Natural reward processing

With repeated drug-use, neural sensitization may occur whereby the drug elicits a potent response in striatal-dopamine neurons coupled with a strong degree of positive reinforcement. At the same time, long-term exposure to drugs significantly attenuates neural responses to intrinsically rewarding stimuli in the environment (e.g., a beautiful landscape, the smile of a baby, a delicious meal). In other words, through neuroadaptation the addicted individual learns to experience reward via self-administration of drugs rather than by enjoying the subtle beauty of the natural environment or the affiliative or health-promoting objects found therein. This re-wiring of reward learning entrenches the drug user in a cycle of drug-taking that serves to maintain the ongoing use of drugs. Indeed, vulnerability to relapse has been attributed to increased incentive salience of drug-cues and decreased salience of intrinsically rewarding stimuli (166, 167). Though pharmacotherapies may provide acute relief to drug addiction-related anhedonia, their effectiveness in facilitating restoration of normal, healthy reward learning remain unknown. Thus, therapies that target reward processes over the long-term, either in the absence of, in adjunct to, or after pharmacotherapies have been discontinued, are very much needed.

By teaching participants to mindfully attend to pleasurable objects, events, and experiences, MBIs may amplify hedonic processing of natural rewards and thereby counter the allostatic effects of addiction on reward neurocircuitry. This form of selective attention to positive experience, known as savoring, is one of the most robust positive emotion regulation strategies (168). During savoring, one not only attends to a broadened diversity and range of sensations and perceptions, but also to the positive emotions elicited by the sensory-perceptual experience. As such, attending to present-moment experience prospectively predicts positive emotion (169). Thus, learning to mindfully savor pleasant events may offset negative affective states that often trigger addictive responses, and restore the salience of naturally occurring and intrinsically rewarding objects and events (e.g., social affiliation, healthy diet and exercise behaviors, engagement with novel and stimulus-rich environments, etc.). Through mindful savoring, MBIs may provide a means of reward replenishment and ultimately reverse the reward deficiency syndrome inherent in addiction – a therapeutic process plausibly important for allaying craving and deterring relapse.

Research suggests that mindfulness training can increase reward experience and positive emotion in both healthy and clinical populations (7). In studies of healthy individuals, mindfully savoring food items increased pleasure from eating (170), and mindfulness training amplified positive stimulus evaluations (171) and increased positive emotional information processing (172). In clinical populations with low positive and high negative affect, MBIs have been shown to be effective means of enhancing positive emotion. Studies demonstrate facilitative effects of mindfulness training on positive affect on patients with major depressive disorder (173), bipolar disorder (174), and HIV (175). Importantly, Geschwind et al. (173) found that Mindfulness-Based Cognitive Therapy increased reward experiences from pleasant daily events among persons in partial remission from major depressive disorder. Insofar as intentional up-regulation of positive emotion is believed to involve increased activation in a predominantly left lateralized prefrontal network that potentiates striatal activation (135, 176), mindfulness-induced positive affectivity may remediate impaired dopaminergic responses in the striatum to hedonic stimuli. Plausibly, MBIs, which also increase left lateralized PFC activity (177), may restore natural reward processes among drug addicted individuals seeking abstinence. The most direct support of this hypothesis stems from recent findings from a RCT of Mindfulness-Oriented Recovery Enhancement for prescription-opioid-misusing chronic pain patients. Participation in 8 weeks of this particular MBI (which specifically focuses on mindful savoring as a key therapeutic process) resulted in significantly enhanced reward responsiveness as indicated by cardiac autonomic responses to positive emotional stimuli presented during a dot probe task. Crucially, the opioid craving-reductive effects of the intervention were statistically mediated by enhancements in reward responsiveness (Garland et al., submitted for publication).

The effects of mindfulness-centered regulation of reward processing might be measured with a positive emotion regulation neuroimaging paradigm, in which participants are instructed to up-regulate positive affective response to intrinsically rewarding stimuli. If mindfulness training fosters reward processing, individuals in recovery from addiction might exhibit enhanced dopaminergic striatal responses to naturally rewarding stimuli coupled with enhanced ratings of stimulus valence. Alternatively, the Effort Expenditure for Rewards Task [EEfRT, (178)] is a task probe of a subject’s willingness to expend effort to receive reward, and theoretically an indirect probe of dopamine-mediated reward processes. In brief, during this task, subjects are provided with a choice period in which they may choose to perform either an easy task for a smaller reward or a hard task for a larger reward. The probability of winning is manipulated and stated to the subject during the choice period. Following the choice period, subjects perform the task and receive feedback about their wins. MBI-related enhancement of reward responsiveness might be indexed by increasing hard task engagement and modifying reaction times on the task.

### Exposure and extinction

Individuals in early recovery from addiction often attempt to suppress craving for drugs and alcohol as a means of maintaining abstinence. However, these suppression attempts often backfire, resulting in depletion of self-control resources (1, 179) and a consequent rebound of substance-related thoughts (50, 51). Critically, attempted avoidance of substance cue-reactivity may prevent extinction learning from occurring, which requires inhibition of conditioned responses in the presence of conditioned stimuli. In contrast, mindfulness training provides an effective alternative to suppressing unwanted substance-related thoughts, emotions, and urges by promoting acceptance of and exposure to these mental experiences. By learning to tolerate aversive psychological events through acceptance rather than avoidance, mindful exposure to substance-related thoughts and cues may prevent the post-suppression rebound effect and facilitate desensitization to conditioned stimuli (78). When engaged over time, this practice might result in extinction learning of previously conditioned associations between substance cue-reactivity and addictive behaviors.

In support of this hypothesis, changes in thought suppression have been shown to partially mediate effects of mindfulness training on alcohol use and drinking consequences (180). Furthermore, Mindfulness-Oriented Recovery Enhancement treatment led to significant reductions in thought suppression which were associated with improved capacity to inhibit drinking urges, decreased alcohol attentional bias, and increased HRV recovery from stress and alcohol cues (7). Relatedly, among a sample of persons in long-term treatment for co-occurring psychiatric and substance use disorders, individuals with higher levels of dispositional mindfulness exhibited less craving for substances and were less likely to develop post-traumatic stress symptoms in response to trauma (66). Thus, MBIs may reduce the tendency to suppress aversive psychological experiences, thereby allowing urges that had been previously suppressed to become accessible to explicit cognitive control. As suppression decreases, controlled cognitive processing can be more effectively deployed to inhibit and counter addictive responses.

The effects of MBIs on cognitive regulation of extinction learning might be measured by combining neuroimaging, self-reported craving, and self-reported emotion regulatory strategy during a drug cue-reactivity paradigm. Pre- and post-mindfulness training, addicts could participate in cue-exposure sessions (e.g., a smoker might be asked to handle cigarettes, ashtrays, and lighters without smoking for a limited period of time, followed by an *ad libitum* smoking session) in which skin conductance, heart rate, and craving responses could be measured throughout. If mindfulness enhances cognitive regulation of extinction learning, cue-elicited skin conductance, heart rate, and self-reported craving would be reduced following mindfulness training relative to an active control intervention, as would drug-use following the cue-exposure session.

Although we have described the aforementioned therapeutic mechanisms of mindfulness-centered regulation as discrete processes linked in a sequential, linear fashion, in actuality they often run in parallel and may be linked in a recursive, self-reinforcing system of positive feedback loops. Figure 2 depicts the hypothesized interactions between these processes and therapeutic targets of MBIs.

## Conclusion

In contrast to mindfulness, which leads to cognitive and behavioral flexibility, addiction may be characterized by *mindlessness*, i.e., habitual or scripted responses that are often deployed automatically without conscious volition or regard for goodness-of-fit with present goals or the socioenvironmental context. Although procurement of many psychoactive substances requires significant planning and intentionality, the appetitive drive that motivates drug seeking may emerge in a context of mindlessness, manifested as obsessional thoughts of using and compulsive urges that seem to arise in an unbidden and intrusive fashion in direct contradiction to rational decision making. Moreover, the behavioral routines involved in the ritual of drug administration can become automated and executed mindlessly in much the same was as other complex repertoires can be engaged without conscious volition by conditioned contextual cues (26). Hence, individuals treated for substance dependence with higher levels of mindlessness tend to experience higher levels of craving (66) and consume larger quantities of addictive substances than their more mindful counterparts (1). These findings suggest that habitual, reflexive responding can confer vulnerability to individuals in recovery. Conversely, greater attention to and awareness of one’s reactions to substance-related cues predicts less substance use among persons in recovery from addiction (111). In light of Tiffany’s (20) proposal that automaticity drives appetitive addictive responses, mindfulness of one’s automatized reactions would presumably allow for greater self-regulation of mindless reactions elicited by drug-cues, and increase proactive cognitive control over substance use.

Addiction involves deleterious neuroplastic changes in frontal-striatal-limbic circuitry that results from chronic drug-use. We hypothesize that this drug-induced neuroplasticity may be remediated through participation in MBIs. Specific knowledge of the neuroplastic alterations underpinning dysregulated circuit-function in addiction may inform treatment development efforts to drive the next generation of MBIs. For example, in light of evidence that dopaminergic salience networks involved in normal human learning and reward become usurped during the addictive process and biased in favor of drug-relevant stimuli, MBIs should be explicitly tailored to address reward processing deficits by emphasizing skills that enhance savoring of natural, non-drug related rewards. Though most current MBIs have underemphasized this potential treatment target, one novel MBI, Mindfulness-Oriented Recovery Enhancement (6–8) places a special emphasis on providing training in mindful savoring as an approach to restoring natural reward responsiveness. Concomitantly, a growing recognition of the role of attentional bias in addiction points to the potential clinical utility of focused attention forms of mindfulness practice as means of strengthening lateral frontal (dlPFC)-parietal networks involved in attentional (re)orienting from drug-cues; in that regard, recent ERP analyses of EEG data suggest that regular, brief mindfulness practice of focused attention on respiratory sensations strengthens electrophysiological indices of enhanced attentional control (181). Conversely, open monitoring forms of mindfulness meditation that target medial frontal (ACC)-parietal-thalamic regulation of striatal circuits might be most useful for generating awareness of cue-elicited activation of drug-use action schemas and could enable the practitioner to regain conscious cognitive control of automatized addictive behavioral routines. Thus, translating findings from the leading edge of neuroscience into the treatment development process may result in ever more specialized and efficacious MBIs targeted to meet the unique challenges of addictions treatment.

Importantly, although various drugs of abuse do share some common neurobiological underpinnings, there is also variability in the circuit-level function associated with different psychoactive agents. Similarly, while addiction to drugs may share overarching neural substrates with some behavioral addictions [e.g., food addiction, (182)], there may be important differences in the functional connectivity or node strength of neural networks involved in these various forms of addiction. Despite these differences, given our assertion that MBIs strengthen domain-general neurocognitive resources that can be used to target common transdiagnostic processes (i.e., automaticity, attentional bias, appraisal, emotion regulation, cue-elicited craving, stress reactivity, and extinction learning) we hypothesize that MBIs would have similar efficacy across a wide range of addictions. In contrast, the efficacy of MBIs may be moderated by key individual differences – an, important understudied area of research crucial to understanding the path from drug initiation to dependence to recovery. For instance, we hypothesize that extant MBIs may be less effective for individuals lacking motivation or readiness to change, because current programs do not integrate motivational components with mindfulness training, and evidence suggests that mindfulness alone may not facilitate readiness to change (Garland et al., submitted for publication). Similarly, MBIs may be more effective for individuals in early to late abstinence as opposed to individuals in active addiction; exposure to ubiquitous drug-related cues and an environment that affords ready access to drugs may promote a more automatized form of drug-use (20) that does not allow a novitiate of mindfulness (whose prefrontally mediated executive functions have atrophied due to years of drug-use) to marshal proactive cognitive control via mindfulness practice. In contrast, inpatient treatment settings may provide respite from cue-elicited craving and contextual triggers of striatally mediated habit responses, and therefore allow a fledgling mindfulness practitioner the opportunity to exercise PFC functionality in a safe environment until it has reached sufficient strength to allow the person to navigate a socio-cultural context beset by stressors and conditioned appetitive stimuli. Thus, mindfulness training may have heterogeneous effects across individuals depending on the natural history and trajectory of their addiction and treatment process.

The conceptual framework we have outlined in this paper may also have utility in developing temporally sequenced descriptions of neurocognitive processes targeted by MBIs. We offer the following speculative, hypothetical account based on our clinical and research experience using MBIs to treat persons diagnosed with substance use disorders. When a recovering addict with a history of using drugs to cope with negative emotions encounters a cue associated with past drug-use episodes while in the context of a stressful environment (e.g., walking past a bar after getting in an argument with a work supervisor), this encounter may activate cortico-limbic-striatal circuits subserving drug-use action schemas. After completing a course in mindfulness training, the addict may become more aware of the automatic addictive habit as it is activated, allowing for top-down regulation of the precipitating negative emotional state and the bottom-up appetitive urge. Specifically, the individual may engage in mindful breathing to first disengage from and then restructure negative cognitive appraisals, thereby reducing limbic (e.g., amygdala) activity, autonomic reactivity, and dysphoric emotions related to the stressor. Concurrently, the individual may become aware of when his attention has been automatically captured by the sight of people drinking in the window of the bar, and, through formal mindfulness practice, activate fronto-parietal mediated attentional networks to disengage and shift focus onto the neutral sensation of respiration. During this process, as sensations of craving arise, the individual may engage in metacognitive monitoring of these sensations, and in so doing, facilitate prefrontal down-regulation of limbic-striatal activation. As mindfulness of craving is sustained over time without drug-use, the sensations of craving may abate, promoting extinction learning to weaken associative linkages between conditioned addiction-related stimuli and the attendant conditioned appetitive response. Once working memory has been cleared of active representations of substance use, the individual may shift attention to savor non-drug related rewards, such as the sense of accomplishment that may arise from successfully resisting the temptation to drink (i.e., self-efficacy), appreciating the beauty of the sunset on the walk home without being clouded by inebriation, or the comforting touch of a loved one upon returning home safe and sober. Through repeated practice of regulating addictive responses and extracting pleasure from life in the absence of substance use, the individual may re-establish healthy dopaminergic tone and foster neuroplasticity in brain areas subserving increased dispositional mindfulness.

Ultimately, mindfulness may facilitate a novel, adaptive response to the canonical “people, places, and things” that tend to elicit addictive behavior as a scripted, habitual reaction. In so doing, the practice of mindfulness may attenuate stress reactivity and suppression while disrupting addictive automaticity, resulting in an increased ability to regulate and recover from addictive urges. The neurocognitive framework we have presented is intended to stimulate future research and facilitate the optimization of MBIs for the treatment of addiction. The tools of modern science have only begun to elucidate the many ways in which mindfulness training targets the risk chain of addiction at the attention-appraisal-emotion interface.

## Conflict of Interest Statement

The authors declare that the research was conducted in the absence of any commercial or financial relationships that could be construed as a potential conflict of interest.
